# Memory reconsolidation impairment by amyloid beta (1–42) and its prevention by non-competitive antagonists of NMDA receptors

**DOI:** 10.3389/fncel.2025.1629492

**Published:** 2025-07-31

**Authors:** A. A. Tiunova, E. A. Diffine, K. V. Anokhin

**Affiliations:** ^1^Institute for Advanced Brain Studies, Lomonosov Moscow State University, Moscow, Russia; ^2^Department of Biology, Lomonosov Moscow State University, Moscow, Russia

**Keywords:** memory, reconsolidation, chicks, β-amyloid, NMDA antagonists, memantine

## Abstract

In a healthy brain, the reactivation of memories under conditions of novelty leads to their labilization and subsequent reconsolidation. However, if plasticity of the nervous system is reduced reconsolidation mechanisms may be disrupted, leading to weakening and loss of existing memory. We hypothesize that such self-degradation of old memory due to its reactivation in the compromised brain may lead to progressive memory loss in Alzheimer’s disease. Preventing memory lability when accessing it, may slow down such engram degradation. To test these hypotheses, we first examined whether beta-amyloid peptide Aβ_1–42_ can impair reconsolidation of memory in one-trial passive avoidance task in young chicks. Next, we examined the possibility to prevent such reminder-associated amnesia by administering a non-competitive N-methyl-D-aspartate (NMDA) receptor antagonist MK-801 prior to memory reactivation. Finally, we compared the memory protecting effects of two non-competitive NMDA antagonists, MK-801 and memantine which is a clinically used medication for treatment of Alzheimer’s disease. We found that administration of Aβ_1–42_ prior to memory reactivation in passive avoidance task in chicks impaired its subsequent reconsolidation. Concurrent systemic injection of MK-801 or memantine prevented this impairment. Our data thus support the hypothesis about the possible role of impaired reconsolidation in the progressive deterioration of old memories in neurodegenerative diseases, particularly in Alzheimer’s disease. This hypothesis offers a new explanation for the protective effects of memantine and suggests the possibility of similar effects with other NMDA receptor antagonists.

## 1 Introduction

Animal models of Alzheimer’s disease primarily study impairments in acquiring new experiences and consolidation of acquired memories (Puzzo et al., 2014; Webster et al., 2014). In this study, we test the hypothesis that β-amyloid (Aβ_1–42_) may affect memory not only by impairing memory consolidation, but also by interfering with its reconsolidation, a process that occurs when a previously consolidated memory is reactivated and becomes temporarily labile (Dudai and Eisenberg, 2004; Dudai, 2006; Nader, 2015; Bellfy and Kwapis, 2020). This hypothesis has important implications for the brain pathology: if memory reconsolidation is vulnerable to Aβ pathology, this may result in gradual degradation of already formed memories in Alzheimer’s disease. If so, preventing the transition of memory into a labile form during its retrieval can be used to slow down its self-degradation in the brain which has diminished reconsolidation mechanisms. This hypothesis can be directly tested in animals with amyloid pathology by pharmacologically preventing memory destabilization during its reactivation.

Pharmacological regulation of memory reconsolidation has been well-studied using learning models in different animal species (Przybyslawski and Sara, 1997; Kida et al., 2002; Kelly et al., 2003; Koh and Bernstein, 2003; Ben Mamou et al., 2006; Crowe et al., 2008). Memory reactivated by a reminder along with blockade of protein synthesis is unable to reconsolidate, resulting in reminder-associated amnesia (Nader et al., 2000; Anokhin et al., 2002; Lopez et al., 2015; Bonin and De Koninck, 2015; Bellfy and Kwapis, 2020). Such amnesia can be prevented by antagonists of NMDA receptors or their subunits injected before memory reactivation (Ben Mamou et al., 2006; Balaban et al., 2014; Bal et al., 2017; Nikitin et al., 2021; Rossato et al., 2023). We recently showed that in young chicks trained in a passive avoidance model, amnesia induced by memory reactivation paired with protein synthesis blockade can be prevented by the non-competitive NMDA receptor antagonist MK-801 (Tiunova et al., 2024).

An important advantage of the passive avoidance learning model in chicks is that it allows for precise timing of training, reactivation, and testing of memory. This temporal resolution is critical for studying phase-specific pharmacological effects on consolidation or reconsolidation. The neural mechanisms underlying memory consolidation and reconsolidation in this model have been extensively studied (Rose, 2000; Crowe et al., 2008). In addition, this model has been proposed as a valuable tool for Alzheimer’s disease research due to the close homology between the amyloid precursor protein (APP) of chick and humans. In chicks, APP plays a critical role in memory consolidation, and disruption of its synthesis results in amnesia (Mileusnic and Rose, 2010).

A single administration of β-amyloid peptides (Aβ_1–42_ or Aβ_12–28_) has previously been used as a model of amyloid pathology in chicks (Mileusnic et al., 2004, 2005; Gibbs et al., 2010; Gibbs, 2015). Administration of Aβ fragments from 24 h before to 15 min after training in the passive avoidance model impaired recall when tested from 35 min to 24 h after training (Gibbs et al., 2010). However, the effects of β-amyloid fragments on memory reconsolidation in chicks has not been previously studied. Accordingly, the first objective of this study was to investigate the effect of β-amyloid on memory reconsolidation. In the second stage, we tested the possibility of preventing reminder-associated retrograde amnesia by administering the NMDA receptor antagonist MK-801 prior to memory reactivation. Finally, we compared the effects of MK-801 and memantine, a drug that is also a non-competitive NMDA receptor antagonist and is used clinically to treat Alzheimer’s disease (Peng et al., 2023; Tang et al., 2023; Karimi Tari et al., 2024).

## 2 Materials and methods

### 2.1 Subjects

The study was carried out in accordance with the recommendations of the Directive 2010/63/EU of the European Parliament and of the Council of the European Union issued 22 September 2010, on the protection of animals, used for scientific purposes (Section 27). The protocol was approved by the Animal Ethics Committee of the Lomonosov Moscow State University.

Domestic chicks (*Gallus gallus domesticus*, Panzirevskaya Black strain) of both sexes were delivered from the Research and Technological Poultry Institute, Moscow Region, on the next day after hatching. The chicks were placed in pairs in metal pens (20 × 25 × 20 cm) with access to food and water and allowed to acclimatize overnight. The experimental room was maintained at 30°C and 12:12 h dark/light cycle. The chicks were taken in the experiments on the following morning, i.e., at the age of 2 days.

In total, 906 chicks were used for the experiments and 823 of them were used in the data analysis. The withdrawal (9.2%) was applied to the chicks that did not peck in the pre-training or training trials and to those which pecked at the aversive bead during the reminder session (see below). Each chick was used only once for training, reminder, and testing.

All behavioral procedures were carried out by a researcher blind to the injected solutions and to which experimental group each chick belonged.

### 2.2 Passive avoidance learning

Passive avoidance learning is based on the innate predisposition of young chicks to try and peck at novel small objects. To stimulate their pecking activity, the chicks were first pre-trained with two 10 s presentations of a 3 mm dry metal bead on a rod, with 5 min interval between the presentations. Only chicks that pecked at the bead (normally over 90% of the total) were included in the experiment. Twenty minutes after the second pre-training, the chicks underwent training with a 2 mm white plastic bead on a rod coated with a bitter substance, methyl anthranilate (Sigma). After pecking at the bead, the chicks exhibited a species-specific disgust reaction (head shaking, beak wiping, distress calls) and afterward avoided pecking an identical but dry bead during subsequent presentations.

### 2.3 Reminder procedure

Two hours post-training, the chicks were presented for 10 s with a dry white bead identical to the training bead. Normally, the chicks did not peck at the bead and displayed typical noticeable avoidance behavior, such as backing away and distress calls (> 80% of the total). This procedure served as reminder that reactivated the memory formed after the training (Anokhin et al., 2002). The response of pecking or avoiding the dry bead was recorded and those that pecked were excluded from further experiments.

### 2.4 Testing

The retention test was given either 4 or 24 h after the training. The testing procedure was identical to the reminder, involving presentation of the same dry white bead. Responses (peck or avoid) were recorded, and a percentage avoidance score was calculated for each experimental group as a proportion of avoiding animals (Tiunova et al., 2024).

To exclude the possibility of a generalized avoidance behavior, 20 min after testing memory for an aversive bead, a neutral bead of a different color was presented. Only the chicks that peck at it and thus distinguished an aversive bead from a neutral one, were included in the analysis.

Avoidance levels between groups of chicks were compared using the two-sided prop.test in R statistics and *post hoc* χ2 test of independence. Differences were considered significant at *P* < 0.05.

### 2.5 Drugs and injections

Amyloid: β-amyloid (Aβ_1–42_, Bachem) was dissolved in DMSO (1 mg/100 μl) and stored at −80°C in aliquots. Immediately before use the aliquots were diluted with physiological saline to the final concentration of Aβ_1–42_ 1 μg/μl. The solution was used within 30 min after preparation to avoid aggregation. The control peptide, scrambled Aβ_1–42_ (Bachem), was prepared in the same way.

Bilateral intracranial injections of Aβ_1–42_ and scrambled Aβ_1–42_ were performed 45 min prior to training or to reminder using a 10 μl Hamilton syringe and a specialized headholder to target the lateral ventricles and adjacent brain areas (Davis et al., 1979). In the dose-dependency experiment each chick of the experimental groups received 0.5–4 μg of the peptide in the volume of 2 μl/hemisphere. Chicks from the control groups received 4 μg of scrambled peptide or physiological saline in the same volume.

MK-801: MK-801 [(+)-MK-801 hydrogen maleate, Sigma] was administered intraperitoneally at a dose of 0.25 mg/kg in 0.1 ml saline, 30 min before the reminder.

Memantine: Memantine hydrochloride (Sigma) was administered intraperitoneally at a dose of 0.73 mg/kg in 0.1 ml saline, 30 min before the reminder.

In chicks, 0.1 ml of 0.1 mM memantine solution was shown to improve memory in weak learning and reactivation of weak memory (Samartgis et al., 2012). A 10-fold higher dose (0.1 ml of 1 mM solution) prevented scopolamine-induced amnesia (Barber and Haggarty, 2010) and enhanced memory in observational learning (Barber and Kimbrough, 2015). Memory was also improved in isolation-induced stress by 0.1 ml of 5 mM solution (Barber et al., 2010). Based on these data, we chose a 0.1 ml of 1 mM memantine (0.73 mg/kg) to test the possibility of preventing reminder-associated memory deficit modeled by Aβ_1–42_.

### 2.6 Experimental groups

In each experiment with memory reactivation there was a control group that received no injections and no reminder (Control). The other controls included groups receiving memory reactivation along with physiological saline or scrambled peptide injection, and groups with β-amyloid injection without the reminder. In the experimental groups the memory was reactivated along with administration of Aβ_1–42_, MK-801, memantine, or their combinations.

## 3 Results

### 3.1 Aβ_1–42_ impairs memory consolidation

**FIGURE 1:**
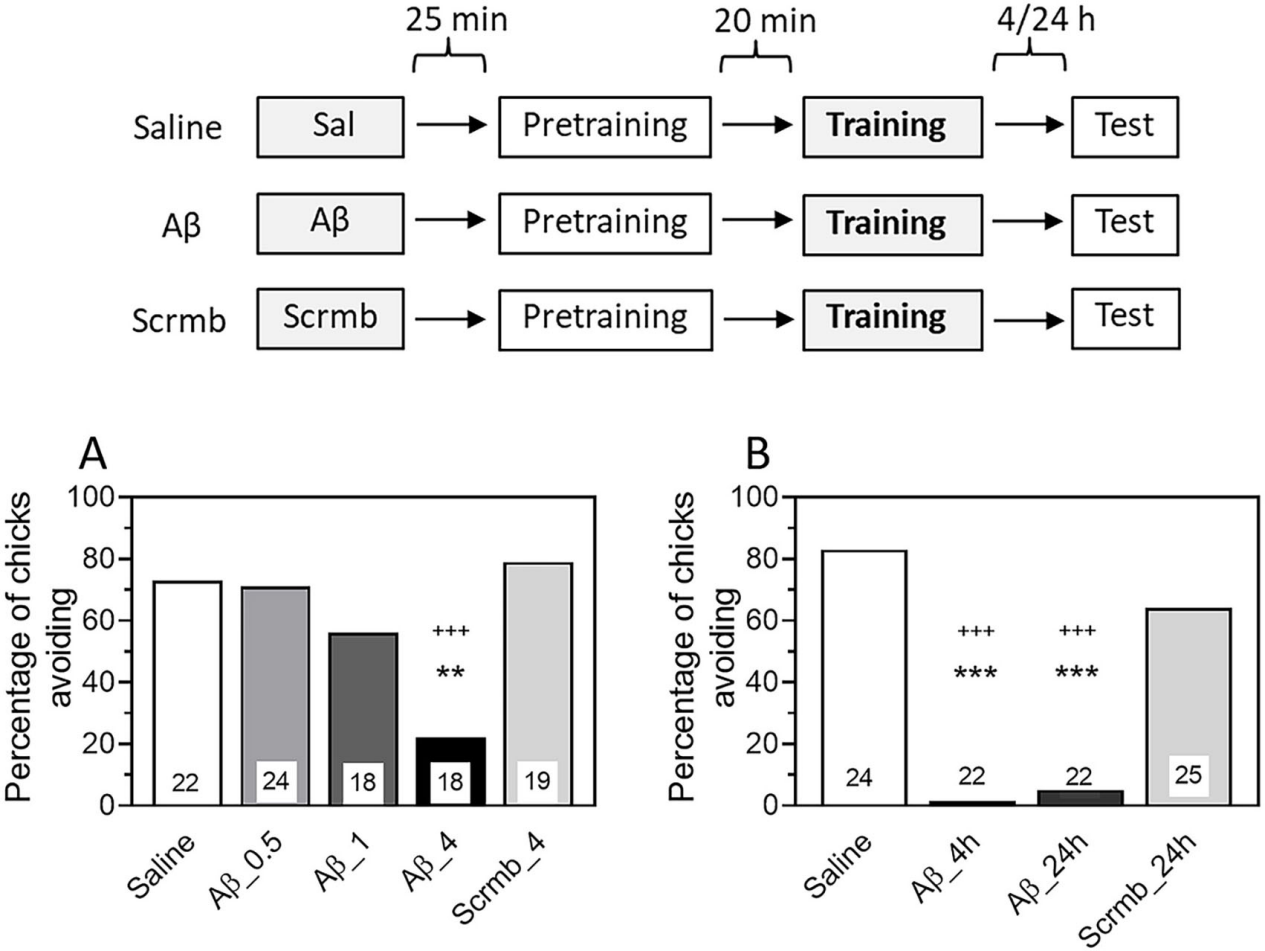
Administration of β-amyloid (1–42) prior to training impairs memory retention. Data are shown as the percentage of chicks showing avoidance. Numbers of chicks in each group are shown in the bars. **(A)** Experimental groups: Saline, pre-training saline injection; Aβ, pre-training injection of Aβ_1–42_ (0.5, 1 or 4 μg); Scrmb, pre-training injection of scrambled peptide (4 μg). Avoidance levels in the retention test 24 h after the training. Injections 45 min pre-training. **P < 0.01 compared to Saline; ^+++^*P* < 0.001compared to Scrmb group. **(B)** Experimental groups: Saline, pre-training saline injection; Aβ_4 h, pre-training injection of Aβ_1–42_ (4 μg), test 4 h after the training; Aβ_24 h, pre-training injection of Aβ_1–42_ (4 μg), test 24 h after the training; Scrmb_24 h, pre-training injection of scrambled peptide (4 μg), test 24 h after the training. ***P < 0.001 compared to Saline; ^+++^*P* < 0.001 compared to Scrmb_24 h group.

In the first experiment, we tested the effect of a single administration of β-amyloid on memory during its formation (consolidation). β-amyloid Aβ_1–42_ was administered 45 min before passive avoidance training in doses ranging from 0.5 to 4 μg. Control groups were administered with scrambled peptide (Scrmb, 4 μg) or saline. Testing 24 h later showed that Aβ_1–42_ at a dose of 4 μg significantly reduced the level of avoidance (Figure 1A, group Aβ/4μg, χ2 = 10.1, *P* < 0.001 compared with the Saline group; χ2 = 11.91, *P* < 0.001 compared with the Scrmb group). These results are consistent with data previously obtained in the same learning model (Mileusnic et al., 2005).

In all subsequent experiments, β-amyloid was administered at a dose of 4 μg. Since the main objective of our work was to investigate the effects of β-amyloid on reactivated memory, we examined whether the effect of Aβ_1–42_ administration would be evident when tested 4 h after training. We found that the avoidance level in the Aβ_1–42_ group was significantly reduced compared with the saline and scrambled peptide groups and was not different from the avoidance behavior in the Aβ group tested 24 h later (Figure 1B, χ2 = 51.06, *P* < 0.001; groups Aβ_4 h and Aβ_24 h: *P* < 0.001 compared with the Saline group and the Scrmb _24 h group). These results are consistent with those previously reported in a passive avoidance model (Gibbs et al., 2010). As shown in this study, administration of Aβ_1–42_ 45 min before training impaired memory when tested as early as 35 min after training, and the memory deficit persisted for at least 24 h.

### 3.2 Aβ_1–42_ impairs memory reconsolidation

β-Amyloid was administered 45 min before memory reactivation by a reminder, i.e., 75 min after training. The Control (no memory reactivation and no injections) and Reminder (Sal/Rem, memory reactivation with saline) groups were used as controls. To exclude the effect of Aβ_1–42_ itself, without association with memory reactivation, an additional control group was administered Aβ_1–42_ 75 min after training, but no reminder was presented (Aβ/NoRem group). To test the specificity of the Aβ_1–42_ peptide effects, another group received scrambled peptide (Scrmb) 45 min before memory reactivation. An additional group of animals was administered scrambled peptide 75 min after training and no reminder was presented.

**FIGURE 2:**
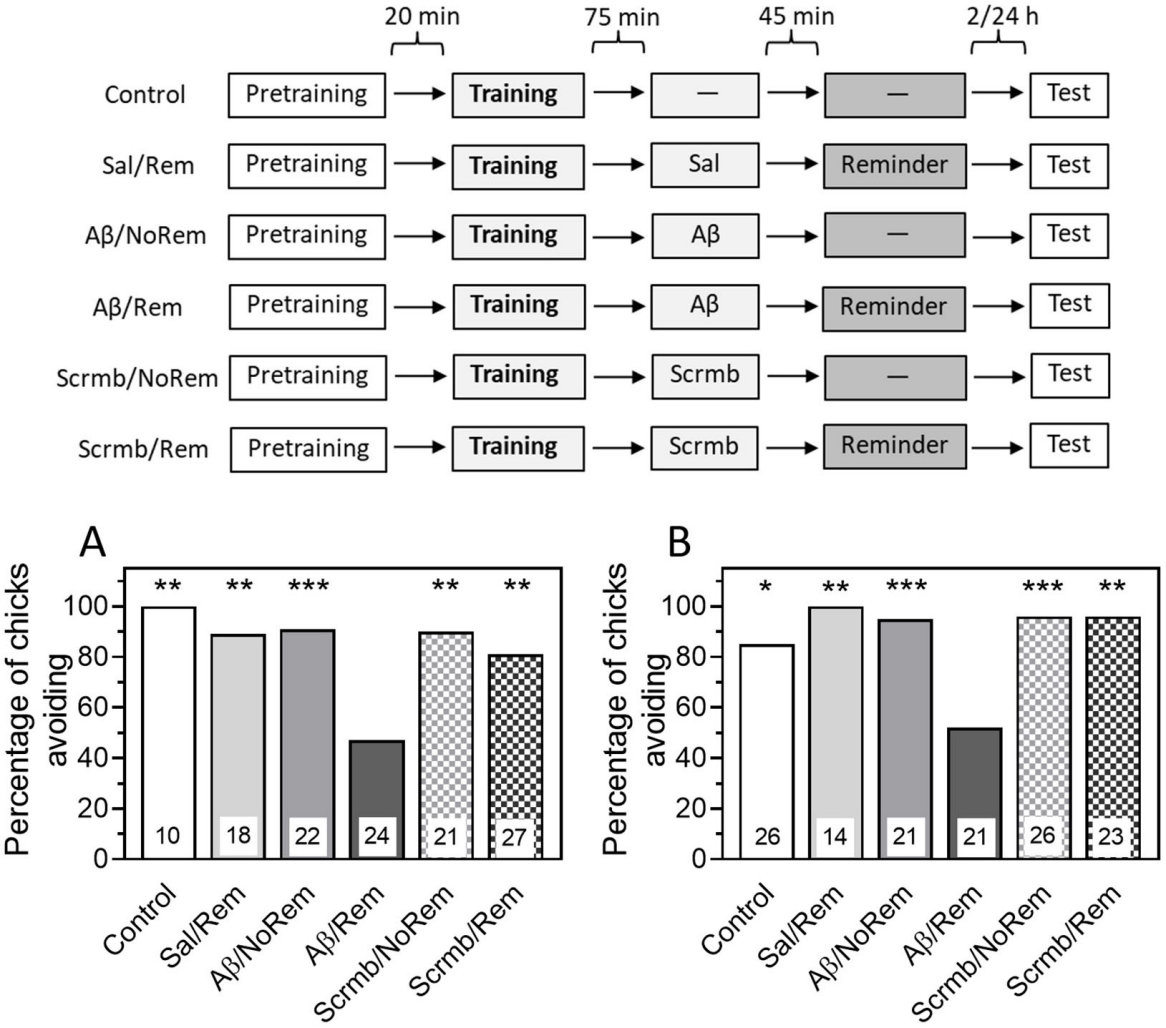
Administration of β-amyloid (1–42) prior to reminder impairs memory retention. Data are shown as the percentage of chicks showing avoidance. Numbers of chicks in each group are shown in the bars. **(A)** Experimental groups: Control, no reminder, no injections; Sal/Rem, reminder coupled with vehicle injection; Aβ/NoRem, injection of Aβ_1–42_, no reminder; Aβ/Rem, injection of Aβ_1–42_ prior to reminder; Scrmb/NoRem, injection of scrambled peptide, no reminder; Scrmb/Rem, injection of scrambled peptide prior to reminder. Avoidance levels in the retention test 4 h after the training. Injections 45 min pre-reminder. **P < 0.01, ***P < 0.001 compared to Aβ/Rem group. **(B)** Experimental groups: Control, no reminder, no injections; Sal/Rem, reminder coupled with vehicle injection; Aβ/NoRem, injection of Aβ_1–42_, no reminder; Aβ/Rem, injection of Aβ_1–42_ prior to reminder; Scrmb/NoRem, injection of scrambled peptide, no reminder; Scrmb/Rem, injection of scrambled peptide prior to reminder. Avoidance levels in the retention test 24 h after the training. Injections 45 min pre-reminder. *P < 0.05, **P < 0.01, ***P < 0.001 compared to Aβ/Rem group.

Administration of Aβ_1–42_ 45 min before memory reactivation by the reminder resulted in a decrease in the level of avoidance when tested 4 h after training (Figure 2A, Aβ/Rem group: *P* < 0.001 compared with Control, χ2 = 8.96, and Sal/Rem, χ2 = 8.7, groups). Administration of Aβ_1–42_ without reminder did not affect memory (Figure 2A, χ2 = 11.19, *P* < 0.001 between Aβ/Rem and Aβ/NoRem groups). Administration of scrambled peptide either with or without reactivation did not affect the level of recall when tested (Figure 2A, Scrmb/Rem and Scrmb/NoRem groups).

Thus, our results show that memory reactivation combined with administration of β-amyloid results in memory impairment, suggesting that Aβ is capable of not only disrupting memory consolidation but also damaging previously acquired memory.

In the next experiment, we tested whether the amnestic effect of β-amyloid persisted when tested 24 h after training. The same groups were used in the experiment as in the previous one. Testing after 24 h showed that the level of avoidance in the group receiving a reminder in the presence of β-amyloid was significantly reduced compared to the control groups that did not receive a reminder or received a reminder in the presence of saline or scrambled peptide (Figure 2B, χ2 = 29.23, *P* < 0.001).

### 3.3 MK-801 prevents amnesia produced by pre-reminder administration of β-amyloid

We recently found that administration of a non-competitive NMDA receptor antagonist MK-801 prevents the reminder-associated amnestic effect of protein synthesis inhibitors on reactivated memory in chicks trained in passive avoidance task (Tiunova et al., 2024).

Therefore, in the next experiment, we tested whether MK-801 could prevent the retention deficit produced by administration of Aβ_1–42_ coupled with memory reactivation.

As in the previous experiments, β-Amyloid was administered 45 min before reminder-induced memory reactivation. MK-801 was administered intraperitoneally at a dose of 0.25 mg/kg in 0.1 ml saline, 30 min before the reminder (Tiunova et al., 2024).

**FIGURE 3:**
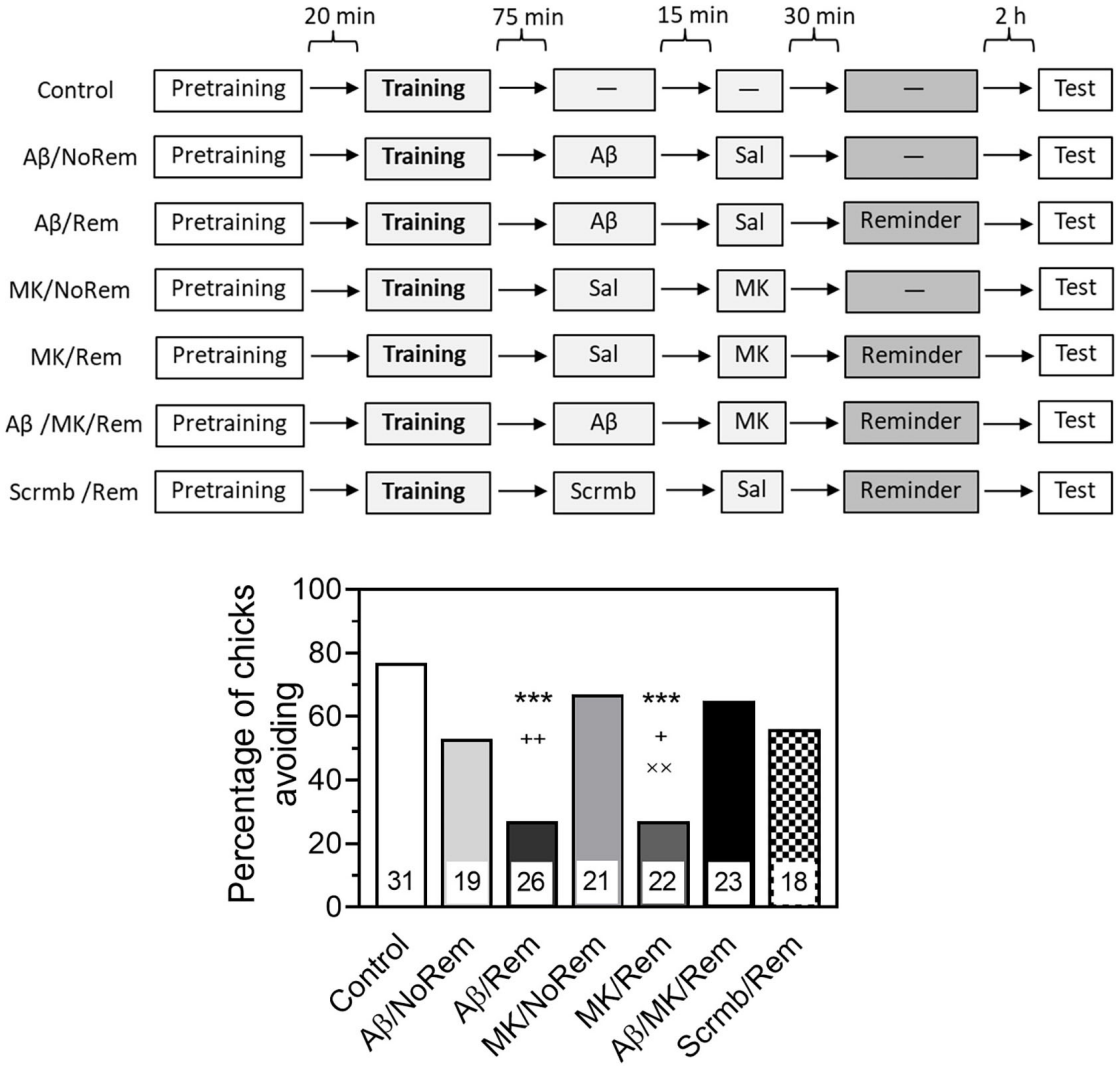
MK-801 prevents reminder-associated amnesia produced by β-amyloid. Experimental groups: Control, no reminder, no injections; Aβ/NoRem, injection of Aβ_1–42_, no reminder; Aβ/Rem, injection of Aβ_1–42_ prior to reminder; MK/NoRem, injection of MK-801, no reminder; MK/Rem, injection of MK-801prior reminder; Aβ/MK/Rem, injection of Aβ_1–42_ and MK-801 prior to reminder; Scrmb/Rem, injection of scrambled peptide prior to reminder. Avoidance levels in the retention test 4 h after the training. Injections of Aβ_1–42_ and scrambled peptide 45 min pre-reminder, MK-801 30 min pre-reminder. ***P < 0.001 compared to the Control group; ^+^*P* < 0.05, ^++^*P* < 0.01 compared to Aβ/MK/Rem group; ^××^*P* < 0.01 compared to MK/NoRem group.

Testing 4 h after training showed that the administration of Aβ_1–42_ in combination with memory reactivation impaired retrieval (Fig. 3, group Aβ/Rem, χ2 = 14.53, *P* < 0.001 compared to the Control group). At the same time, the administration of Aβ_1–42_ alone, without memory reactivation, did not lead to any notable decrease in the level of avoidance (Figure 3, group Aβ/NoRem). Memory reactivation along with the introduction of a scrambled peptide also had no significant effect on the level of avoidance during testing (Figure 3, group Scrmb/Rem). The administration of MK-801 produced an effect similar to the effect of β-amyloid. In combination with memory reactivation, MK-801 significantly impaired memory (Figure 3, group MK/Rem, χ2 = 13.17, *P* < 0.001 compared to the Control group). Administration of MK-801 without memory reactivation did not affect the level of avoidance (Figure 3, MK/NoRem group, χ2 = 6.7, *P* < 0.01 compared with MK/Rem group).

In the group that received a reminder combined with both β-amyloid and MK-801, the level of avoidance was significantly higher than in the groups that received a reminder in combination with β-amyloid alone or MK-801 alone (Figure 3, Aβ/MK/Rem group, *P* < 0.01 compared with Aβ/Rem group, *P* < 0.05 compared with MK/Rem group).

Thus, while β-amyloid and MK-801 alone impaired the retrieval of reactivated memory, no memory impairment was observed when both substances were administered.

### 3.4 Memantine prevents amnesia produced by pre-reminder administration of β-amyloid

**FIGURE 4:**
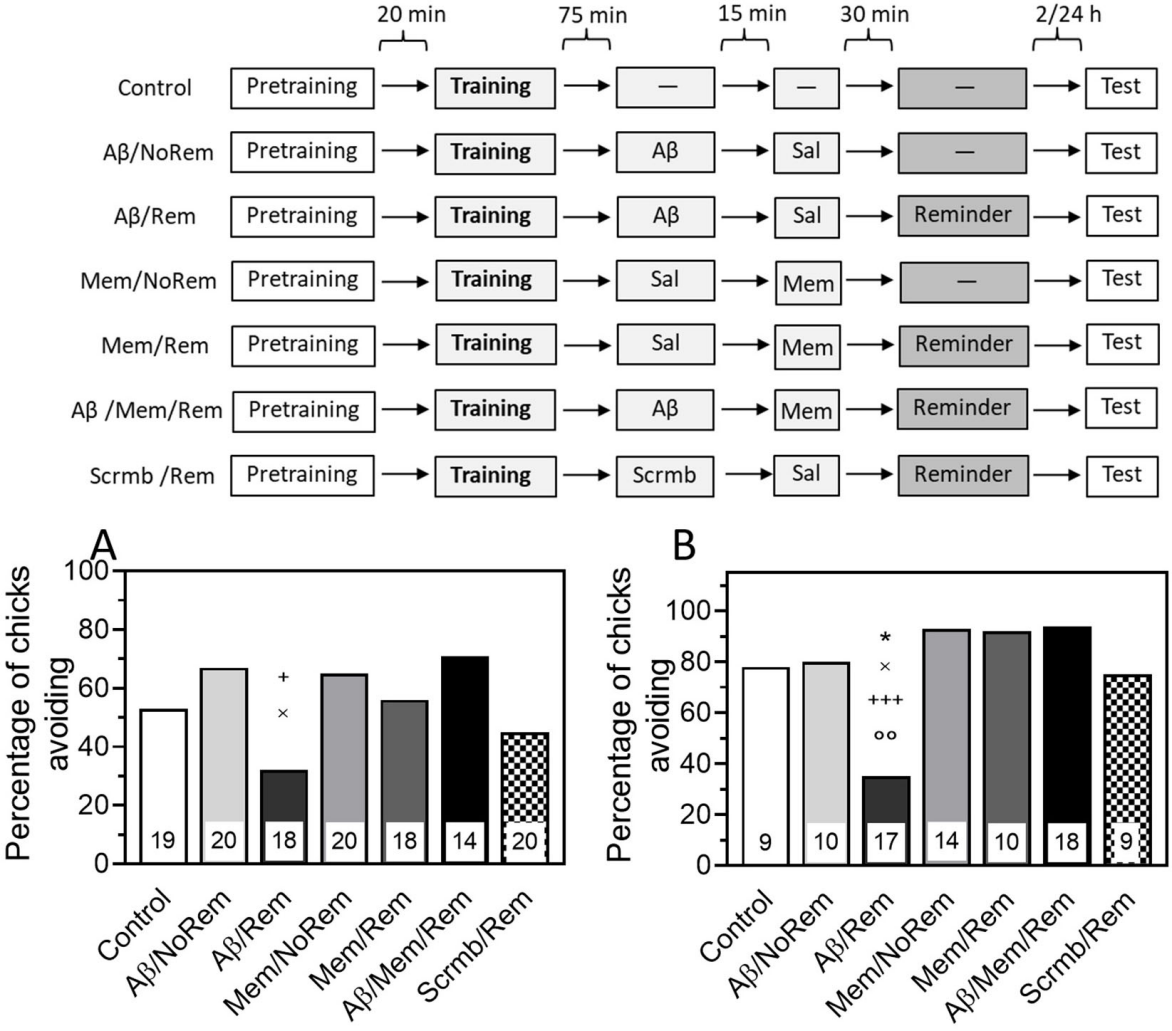
**(A)** Memantine prevents reminder-associated amnesia produced by β-amyloid. **(A)** Experimental groups: Control, no reminder, no injections; Aβ/NoRem, injection of Aβ_1–42_, no reminder; Aβ/Rem, injection of Aβ_1–42_ prior to reminder; Mem/NoRem, injection of memantine, no reminder; Mem/Rem, injection of memantine prior reminder; Aβ/Mem/Rem, injection of Aβ_1–42_ and memantine prior to reminder; Scrmb/Rem, injection of scrambled peptide prior to reminder. Avoidance levels in the retention test 4 h after the training. Injections of Aβ_1–42_ and scrambled peptide 45 min pre-reminder, memantine 30 min pre-reminder. ^+^*P* < 0.05 compared to Aβ/Mem/Rem group; ^×^*P* < 0.05 compared to Aβ/NoRem group. **(B)** Experimental groups: Control, no reminder, no injections; Aβ/NoRem, injection of Aβ_1–42_, no reminder; Aβ/Rem, injection of Aβ_1–42_ prior to reminder; Mem/NoRem, injection of memantine, no reminder; Mem/Rem, injection of memantine prior reminder; Aβ/Mem/Rem, injection of Aβ_1–42_ and memantine prior to reminder; Scrmb/Rem, injection of scrambled peptide prior to reminder. Avoidance levels in the retention test 24 h after the training. Injections of Aβ_1–42_ and scrambled peptide 45 min pre-reminder, memantine 30 min pre-reminder. *P < 0.05 compared to the Control group; ^×^*P* < 0.05 compared to Aβ/NoRem group; ^+++^*P* < 0.001 compared to Aβ/Mem/Rem group; *^oo^P* < 0.01 compared to Scrmb/Rem group.

To test the ability of memantine to prevent amnesia caused by memory reactivation combined with β-amyloid, memantine was administered 30 min before the reminder. Testing 4 h after training showed that memantine administration alone or paired with memory reactivation had no effect on memory. The level of avoidance in the memantine-treated groups did not differ significantly from the level of avoidance in the control group, which did not receive either a reminder or injections (Figure 4A, Mem/Rem and Mem/NoRem groups).

As in previous experiments, β-amyloid administration 75 min after training did not affect memory recall during testing, and the level of avoidance in this group did not differ from the control (Figure 4A, Aβ/NoRem group). At the same time, memory reactivation along with Aβ_1–42_ administration significantly reduced the level of avoidance during testing (Figure 4A, Aβ/Rem group, χ2 = 4.91, *P* < 0.05 compared with Aβ/NoRem group). Reminder in combination with scrambled peptide did not affect the level of avoidance during testing (Figure 4A, Scrmb/Rem group).

Administration of memantine before memory reactivation paired with β-amyloid prevented the amnestic effect: the level of avoidance in the group receiving the reminder together with both β-amyloid and memantine was significantly higher than in the group receiving the reminder in combination with β-amyloid only, and did not differ from the level of avoidance in the control groups (Figure 4A, Aβ/Mem/Rem group, χ2 = 5.12, *P* < 0.05 compared with Aβ/Rem group).

The next experiment tested whether the protective effects of memantine would persist when tested 24 h after training. As with testing 4 h after training, administration of β-amyloid in combination with memory reactivation significantly reduced the level of avoidance (Figure 4B, Aβ/Rem group, χ2 = 4.25, *P* < 0.05 compared with the Control group, χ2 = 10.9, *P* < 0.01 compared with the Scrmb/Rem group). The level of avoidance after administration of Aβ_1–42_ without memory reactivation did not differ from the level of avoidance in the Control group and was significantly higher than in the group receiving Aβ_1–42_ in combination with a reminder (Figure 4B, Aβ/NoRem group, χ2 = 5.04, *P* < 0.05 compared with the Aβ/Rem group). Memantine administration did not affect memory either by itself or in combination with memory reactivation (Figure 4B, Mem/Rem and Mem/NoRem groups). At the same time, memantine administration prevented memory impairment caused by combination of its reactivation with Aβ administration (Figure 4B, Aβ/Mem/Rem group, χ2 = 13.58, *P* < 0.001 compared with Aβ/Rem group).

## 4 Discussion

The present study was based on the hypothesis of self-degradation of already consolidated memory in Alzheimer’s disease due to impairment of its reconsolidation mechanisms in the brain damaged by amyloid pathology. If this hypothesis is correct, then it is possible to slow down the progressive memory loss in Alzheimer’s disease by preventing the destabilization of old memories during their reactivation.

To test these two hypotheses, we first investigated the effect of a single intraventricular injection of β-amyloid Aβ_1–42_ on reactivated memory in the passive avoidance model in young chicks. The ability of this peptide to disrupt memory consolidation in this model has been already shown (Mileusnic et al., 2004, 2005; Gibbs et al., 2009, 2010; Gibbs, 2015). We also studied the effects of different doses of Aβ 45 min before training, and our results showed that Aβ_1–42_ at a dose of 4 μg disrupted memory when tested after 4 and 24 h, which is consistent with previously obtained data. This dose and time of administration were used in further experiments to test the effect of β-amyloid on reactivated memory.

We found that administration of Aβ_1–42_ 45 min before the reminder led to a recall deficit in the test 4 h after memory reactivation. Moreover, the effect of β-amyloid on reactivated memory persisted for 24 h. Our data are consistent with findings from mammalian studies on the vulnerability of memory during reconsolidation in the amyloid-compromised brain. In 5XFAD transgenic mice, a model of Alzheimer’s disease, memory reactivation following fear conditioning resulted in a significant impairment of previously consolidated memory compared to both wild-type controls and 5XFAD mice that were not re-exposed to the memory cue (Ohno, 2009). These results indicate that in the presence of amyloid pathology, the reconsolidation process is impaired, resulting in the weakening of previously established memories. Similarly, in rats, injection of the Aβ_25–35_ peptide during the reactivation of an object recognition memory, impaired its subsequent retrieval (Álvarez-Ruíz and Carrillo-Mora, 2013), providing further evidence that amyloid interferes with the reconsolidation process. APP/PS1 mice with Alzheimer’s disease trained in a water maze at 3 months of age and tested at 7 months showed worse memory than wild-type animals (Rai et al., 2020). However, if they were “overtrained,” i.e., pre-trained at 2 months of age, their performance in the test at 7 months was as good as that of the control group. The authors argue that this improvement involved reconsolidation processes that were impaired in Alzheimer’s disease but could be enhanced by overtraining. These mammalian results are consistent with our data in the chick model, where memory impairment occurs only if Aβ_1–42_ is administered at the time of memory reactivation, but not without it.

Memory reactivation combined with administration of amnestic agents in some cases leads only to transient amnesia, and over time the memory is spontaneously recovered (Litvin and Anokhin, 2000; Milekic and Alberini, 2002; Lattal and Abel, 2004; Salinska et al., 2004; Power et al., 2006; Prado-Alcalá et al., 2006). Studies in a passive avoidance model in chicks have shown that memory reactivation following administration of antagonists of glutamate NMDA, AMPA, and metabotropic receptors (AP5, CNQX, MPEP), cyclin-dependent kinase 5 (roscovitine), RNA synthesis inhibitors (DRB), and inhibitors of protein synthesis or glycosylation (anisomycin, cycloheximide, 2-deoxygalactose) resulted in memory deficits when tested up to 4 h after reactivation, and in all cases no deficits were observed when tested 24 h later (Litvin and Anokhin, 2000; Anokhin et al., 2002; Summers et al., 2003; Salinska, 2006; Sherry and Crowe, 2008a,b; Sherry et al., 2010). In contrast, the administration of these agents during training leads to development of permanent amnesia. Thus, the effect of antagonists on consolidation of memory differs from their effect on the reconsolidation of reactivated memory. In contrast to these data, administration of amyloid peptide (Aβ_1–42_) before memory reactivation in our experiments resulted in retrieval deficits even 24 h later, indicating that its effects differ from those of other amnestic agents.

Next, we investigated the possibility of preventing amnesia produced by administration of β-amyloid paired with memory reactivation. We tested whether the NMDA receptor antagonist MK-801 would be able to prevent amnesia caused by administration of Aβ_1–42_ during memory reactivation. This possibility was partly suggested by our previous data on the ability of MK-801 to prevent reminder-associated amnesia caused by protein synthesis inhibitors. Indeed, as our experiments showed, administration of MK-801 to chicks that received Aβ_1–42_ paired with the reminder had a protective effect, preventing memory deficit.

In addition to the amnesic effects MK-801 in doses above 0.1 mg/kg causes hyperlocomotion, stereotypy, ataxia, and anxiolytic/anxiogenic behavior (Janus et al., 2023). However, in chicks MK-801 was administered intraperitoneally without visible side effects at doses up to 0.4 mg/kg (Burchuladze and Rose, 1992; Bullock et al., 1993; Freeman and Rose, 1995; Tiunova et al., 2020). In the present study, we administered MK-801 in a dose of 0.25 mg/kg without noticeable behavioral changes. In our experimental conditions hyperlocomotion and anxiolytic effects, if present, would have resulted in an increase in the number of chicks pecking during the reminder, which was not observed. Ataxia, if present, would have been noticeable as unsuccessful pecking attempts, but we did not observe such impairments. Anxiogenic effects, if present, would have resulted in more distress and fear calls–no such effects were noticed. Our data also show that the reminder procedure influenced subsequent testing (Figure 3, MK/Rem and MK/NoRem groups), thereby confirming that the chicks were able to pay attention to the bead during the reminder procedure.

Normally, the effects of endogenous β-amyloid and NMDA receptors (NMDARs) appear to interact in several ways. On the one hand, Aβ synthesis requires the activity of extra synaptic NMDA receptors containing the NR2B unit, and, in turn, an increase in Aβ concentration enhances their activity (Li and Selkoe, 2020; Babaei, 2021; Cheng et al., 2021). In addition, soluble Aβ oligomers impair glutamate reuptake by neurons and astrocytes, which also increases the activity of extrasynaptic NMDA receptors (Li and Selkoe, 2020; Li and Stern, 2022). On the other hand, soluble Aβ forms suppress the functions of synaptic NMDA receptors and enhance their internalization, thereby disrupting the balance of the glutamatergic network (Babaei, 2021; Zhang et al., 2022). Hyperactivation of extrasynaptic NMDA receptors leads to excessive Ca^2+^ entry, which triggers a chain of toxic reactions leading to cell membrane destruction, disruption of synaptic transmission, and cell death. The ability to prevent this chain is usually attributed to the action of the NMDA receptor antagonist memantine, used in the treatment of Alzheimer’s disease (Karimi Tari et al., 2024; Nystuen et al., 2024).

However, our work was based on a different hypothesis of the protective effect of NMDAR antagonists on reactivated memory, namely their ability to prevent engram labilization. This hypothesis is supported by the literature (Ben Mamou et al., 2006; Balaban et al., 2014; Bal et al., 2017; Nikitin et al., 2021; Rossato et al., 2023) and our previous results (Tiunova et al., 2024) showing that NMDAR antagonists prevent reminder-associated amnesia regardless of the agent that caused the impairment of reactivated memory. Accordingly, we tested the ability of memantine to prevent amnesia induced by memory reactivation in the presence of Aβ_1–42_.

Systemic administration of memantine 30 min before a reminder prevented the amnesia induced by β-amyloid associated with memory reactivation when tested 4 or 24 h after training. The effect of memantine was comparable to that of MK-801, except that memantine itself, unlike MK-801, did not impair reactivated memory. A possible explanation could be that memantine has lower affinity to NMDAR compared to MK-801 (Cheng et al., 2021). In addition, the effect of memantine on NMDARs demonstrates an order of magnitude faster kinetics than the effect of MK-801 (Rogawski and Wenk, 2003). Additionally, although we could not directly compare the doses of these two antagonists on NMDARs, the doses of memantine we used were significantly lower than those used in clinical settings (Danysz and Parsons, 2003; Parsons et al., 2007; Karimi Tari et al., 2024).

In addition to NMDA receptor antagonist properties, memantine has been shown to enhance cholinergic signaling (Tang et al., 2023). This ability may also contribute to its protective effect on memory by preventing acetylcholine deficiency - one of the main neuropathological features of Alzheimer’s disease. In rats, memantine prevented the loss of cholinergic innervation to the neocortex induced by Aβ_1–42_ (Nyakas et al., 2011). It also reverses scopolamine-induced learning deficits in mice, chicks, and rats (Drever et al., 2007; Barber and Haggarty, 2010; Bali et al., 2019). Since cholinergic transmission is involved in passive avoidance learning in chicks (Rose et al., 1980; Zhao et al., 1997; Mezey et al., 1999), the effects of memantine on the cholinergic system may also have contributed to memory protection in our experiments.

Thus, our results demonstrate a reconsolidation-related mechanism of Aβ-induced amnesia. The fact that memory impairment occurred only when Aβ was administered before the reminder, but not without such reactivation, strongly suggests that Aβ targets the destabilization phase that precedes memory reconsolidation. This is consistent with the notion that memories become transiently unstable after reactivation and require NMDA-dependent restabilization, a process vulnerable to pathological interference. In contrast to consolidation deficits that affect newly formed memories, our data indicate that even well-established memories can be disrupted by reconsolidation impairment in Aβ-compromised brains.

Overall, our data are consistent with the hypothesis that impaired reconsolidation may play a role in the weakening of old consolidated memory in neurodegenerative diseases such as Alzheimer’s disease. In addition to providing another explanation for the therapeutic effect of memantine, this hypothesis also suggests the possibility of a similar effect of other NMDA receptor antagonists, including those used or tested clinically for other purposes (e.g., riluzole, amantadine, neramexane). Testing the possibility of protecting old memory from reactivation-associated impairment with these and other NMDA receptor antagonists may open up a promising new approach for the treatment of neurodegenerative diseases.

## Data Availability

The raw data supporting the conclusions of this article will be made available by the authors, without undue reservation.

## References

[B1] Álvarez-Ruíz Y. Carrillo-Mora P. (2013). Amyloid beta 25-35 impairs reconsolidation of object recognition memory in rats and this effect is prevented by lithium carbonate. *Neurosci. Lett.* 548 79–83. 10.1016/j.neulet.2013.06.003 23774478

[B2] Anokhin K. V. Tiunova A. A. Rose S. P. R. (2002). Reminder effects - reconsolidation or retrieval deficit? Pharmacological dissection with protein synthesis inhibitors following reminder for a passive-avoidance task in young chicks. *Eur. J. Neurosci.* 15 1759–1765. 10.1046/j.1460-9568.2002.02023.x 12081655

[B3] Babaei P. (2021). NMDA and AMPA receptors dysregulation in Alzheimer’s disease. *Eur. J. Pharmacol.* 908:174310. 10.1016/j.ejphar.2021.174310 34265291

[B4] Bal N. V. Rysakova M. P. Vinarskaya A. K. Ivanova V. Zuzina A. B. Balaban P. M. (2017). Cued memory reconsolidation in rats requires nitric oxide. *Eur. J. Neurosci.* 45 643–647. 10.1111/ejn.13503 27987370

[B5] Balaban P. M. Roshchin M. Timoshenko A. K. Gainutdinov K. L. Bogodvid T. K. Muranova L. N. (2014). Nitric oxide is necessary for labilization of a consolidated context memory during reconsolidation in terrestrial snails. *Eur. J Neurosci.* 40 2963–2970. 10.1111/ejn.12642 24910164

[B6] Bali Z. K. Bruszt N. Tadepalli S. A. Csurgyók R. Nagy L. V. Tompa M. (2019). Cognitive enhancer effects of low memantine doses are facilitated by an alpha7 nicotinic acetylcholine receptor agonist in scopolamine-induced amnesia in rats. *Front. Pharmacol.* 10:73. 10.3389/fphar.2019.00073 30804787 PMC6371842

[B7] Barber T. A. Haggarty M. K. (2010). Memantine ameliorates scopolamine-induced amnesia in chicks trained on taste-avoidance learning. *Neurobiol. Learn. Mem.* 93 540–545. 10.1016/j.nlm.2010.02.001 20170739

[B8] Barber T. A. Kimbrough T. N. (2015). Memantine improves observational learning in day-old chicks. *Behav. Pharmacol.* 26 407–410. 10.1097/FBP.0000000000000130 25738760

[B9] Barber T. A. Meyers R. A. McGettigan B. F. (2010). Memantine improves memory for taste-avoidance learning in day-old chicks exposed to isolation stress. *Pharmacol. Biochem. Behav.* 95 203–208. 10.1016/j.pbb.2010.01.006 20100505

[B10] Bellfy L. Kwapis J. L. (2020). Molecular mechanisms of reconsolidation-dependent memory updating. *Int. J. Mol. Sci.* 21:6580. 10.3390/ijms21186580 32916796 PMC7555418

[B11] Ben Mamou C. Gamache K. Nader K. (2006). NMDA receptors are critical for unleashing consolidated auditory fear memories. *Nat. Neurosci.* 9 1237–1239. 10.1038/nn1778 16998481

[B12] Bonin R. P. De Koninck Y. (2015). Reconsolidation and the regulation of plasticity: Moving beyond memory. *Trends Neurosci.* 38 336–344. 10.1016/j.tins.2015.04.007 25987442

[B13] Bullock S. Rose S. P. Pearce B. Potter J. (1993). Training chicks on a passive avoidance task modulates glutamate-stimulated inositol phosphate accumulation. *Eur. J. Neurosci*. 5, 43–48. 10.1111/j.1460-9568.1993.tb00203.x 7505163

[B14] Burchuladze A. A. Rose S. P. R. (1992). Memory formation in day-old chicks requires NMDA but not non-NMDA glutamate receptors. *Eur. J. Neurosci.* 4 533–538. 10.1111/j.1460-9568.1992.tb00903.x 12106339

[B15] Cheng Y. J. Lin C. H. Lane H. Y. (2021). Involvement of cholinergic, adrenergic, and glutamatergic network modulation with cognitive dysfunction in Alzheimer’s disease. *Int. J. Mol. Sci.* 22:2283. 10.3390/ijms22052283 33668976 PMC7956475

[B16] Crowe S. F. Sherry J. M. Hale M. W. (2008). Remembering that things have changed: A review of the cellular mechanisms of memory re-consolidation in the day-old chick. *Brain Res. Bull.* 76 192–197. 10.1016/j.brainresbull.2008.02.020 18498931

[B17] Danysz W. Parsons C. G. (2003). The NMDA receptor antagonist memantine as a symptomatological and neuroprotective treatment for Alzheimer’s disease: Preclinical evidence. *Int. J. Geriatr. Psychiatry* 18 S23–S32. 10.1002/gps.938 12973747

[B18] Davis J. L. Masouka D. T. Gerbrandt D. T. Cherkin A. (1979). Autoradiographic distribution of L-proline in chick after intracerebral injection. *Physiol. Behav.* 22 177–184. 10.1016/0031-9384(79)90233-6 482410

[B19] Drever B. D. Anderson W. G. Johnson H. O’Callaghan M. Seo S. Choi D. Y. (2007). Memantine acts as a cholinergic stimulant in the mouse hippocampus. *J. Alzheimers Dis.* 12 319–333. 10.3233/jad-2007-12405 18198419

[B20] Dudai Y. (2006). Reconsolidation: The advantage of being refocused. *Curr. Opin. Neurobiol.* 16 174–178. 10.1016/j.conb.2006.03.010 16563730

[B21] Dudai Y. Eisenberg M. (2004). Rites of passage of the engram: Reconsolidation and the lingering consolidation hypothesis. *Neuron* 44 93–100. 10.1016/j.neuron.2004.09.003 15450162

[B22] Freeman F. M. Rose S. P. (1995). MK-801 blockade of Fos and Jun expression following passive avoidance training in the chick. *Eur. J. Neurosci*. 7, 563–569. 10.1111/j.1460-9568.1995.tb00661.x 7620608

[B23] Gibbs M. (2015). Reflections on glycogen and β-amyloid: Why does glycogenolytic β2-adrenoceptor stimulation not rescue memory after β-amyloid? *Metab. Brain Dis.* 30 345–352. 10.1007/s11011-014-9563-y 24810634

[B24] Gibbs M. E. Gibbs Z. Hertz L. (2009). Rescue of Abeta(1-42)-induced memory impairment in day-old chick by facilitation of astrocytic oxidative metabolism: Implications for Alzheimer’s disease. *J. Neurochem.* 109 230–236. 10.1111/j.1471-4159.2009.05800.x 19393032

[B25] Gibbs M. E. Maksel D. Gibbs Z. Hou X. Summers R. J. Small D. H. (2010). Memory loss caused by beta-amyloid protein is rescued by a beta(3)-adrenoceptor agonist. *Neurobiol. Aging* 31 614–624. 10.1016/j.neurobiolaging.2008.05.018 18632189

[B26] Janus A. Lustyk K. Pytka K. (2023). MK-801 and cognitive functions: Investigating the behavioral effects of a non-competitive NMDA receptor antagonist. *Psychopharmacology* 240 2435–2457. 10.1007/s00213-023-06454-z 37725119 PMC10640442

[B27] Karimi Tari P. Parsons C. G. Collingridge G. L. Rammes G. (2024). Memantine: Updating a rare success story in pro-cognitive therapeutics. *Neuropharmacology* 244:109737. 10.1016/j.neuropharm.2023.109737 37832633

[B28] Kelly A. Laroche S. Davis S. (2003). Activation of mitogen-activated protein kinase/extracellular signal-regulated kinase in hippocampal circuitry is required for consolidation and reconsolidation of recognition memory. *J. Neurosci.* 23 5354–5360. 10.1523/JNEUROSCI.23-12-05354.2003 12832561 PMC6741214

[B29] Kida S. Josselyn S. A. de Ortiz S. P. Kogan J. H. Chevere I. Masushige S. (2002). CREB required for the stability of new and reactivated fear memories. *Nat. Neurosci.* 5 348–355. 10.1038/nn819 11889468

[B30] Koh M. T. Bernstein I. L. (2003). Inhibition of protein kinase A activity during conditioned taste aversion retrieval: Interference with extinction or reconsolidation of a memory? *Neuroreport* 14 405–407. 10.1097/00001756-200303030-00021 12634492

[B31] Lattal K. M. Abel T. (2004). Behavioral impairments caused by injections of the protein synthesis inhibitor anisomycin after contextual retrieval reverse with time. *Proc. Natl. Acad. Sci. U S A.* 101 4667–4672. 10.1073/pnas.0306546101 15070775 PMC384804

[B32] Li S. Selkoe D. J. (2020). A mechanistic hypothesis for the impairment of synaptic plasticity by soluble Aβ oligomers from Alzheimer’s brain. *J. Neurochem.* 154 583–597. 10.1111/jnc.15007 32180217 PMC7487043

[B33] Li S. Stern A. M. (2022). Bioactive human Alzheimer brain soluble Aβ: Pathophysiology and therapeutic opportunities. *Mol. Psychiatry* 27 3182–3191. 10.1038/s41380-022-01589-5 35484241

[B34] Litvin O. O. Anokhin K. V. (2000). Mechanisms of memory reorganization during retrieval of acquired behavioral experience in chicks: The effects of protein synthesis inhibition in the brain. *Neurosci. Behav. Physiol.* 30 671–678. 10.1023/a:1026698700139 11127794

[B35] Lopez J. Gamache K. Schneider R. Nader K. (2015). Memory retrieval requires ongoing protein synthesis and NMDA receptor activity-mediated AMPA receptor trafficking. *J. Neurosci.* 35 2465–2475. 10.1523/jneurosci.0735-14.2015 25673841 PMC6605616

[B36] Mezey S. Székely A. D. Bourne R. C. Kabai P. Csillag A. (1999). Changes in binding to muscarinic and nicotinic cholinergic receptors in the chick telencephalon, following passive avoidance learning. *Neurosci. Lett.* 270 75–78. 10.1016/s0304-3940(99)00472-3 10462101

[B37] Milekic M. H. Alberini C. M. (2002). Temporally graded requirement for protein synthesis following memory reactivation. *Neuron* 36 521–525. 10.1016/s0896-6273(02)00976-5 12408853

[B38] Mileusnic R. Rose S. (2010). The chick as a model for the study of the cellular mechanisms and potential therapies for Alzheimer’s disease. *Int. J. Alzheimer’s Dis.* 2010:180734. 10.4061/2010/180734 20721285 PMC2915614

[B39] Mileusnic R. Lancashire C. L. Rose S. P. (2004). The peptide sequence Arg-Glu-Arg, present in the amyloid precursor protein, protects against memory loss caused by A beta and acts as a cognitive enhancer. *Eur. J. Neurosci.* 19 1933–1938. 10.1111/j.1460-9568.2004.03276.x 15078567

[B40] Mileusnic R. Lancashire C. L. Rose S. P. (2005). Amyloid precursor protein: From synaptic plasticity to Alzheimer’s disease. *Ann. N Y Acad. Sci.* 1048 149–165. 10.1196/annals.1342.014 16154929

[B41] Nader K. (2015). Reconsolidation and the dynamic nature of memory. *Cold Spring Harb. Perspect. Biol.* 7:a021782. 10.1101/cshperspect.a021782 26354895 PMC4588064

[B42] Nader K. Schafe G. E. Le Doux J. E. (2000). Fear memories require protein synthesis in the amygdala for reconsolidation after retrieval. *Nature* 406 722–726. 10.1038/35021052 10963596

[B43] Nikitin V. P. Kozyrev S. A. Solntseva S. V. Nikitin P. V. (2021). Protein synthesis inhibitor administration before a reminder caused recovery from amnesia induced by memory reconsolidation impairment with NMDA glutamate receptor antagonist. *Brain Res. Bull.* 171 44–55. 10.1016/j.brainresbull.2021.03.008 33722648

[B44] Nyakas C. Granic I. Halmy L. G. Banerjee P. Luiten P. G. (2011). The basal forebrain cholinergic system in aging and dementia. Rescuing cholinergic neurons from neurotoxic amyloid-β42 with memantine. *Behav. Brain Res.* 221 594–603. 10.1016/j.bbr.2010.05.033 20553766

[B45] Nystuen K. L. McNamee S. M. Akula M. Holton K. M. DeAngelis M. M. Haider N. B. (2024). Alzheimer’s disease: Models and molecular mechanisms informing disease and treatments. *Bioengineering (Basel)* 11:45. 10.3390/bioengineering11010045 38247923 PMC10813760

[B46] Ohno M. (2009). Failures to reconsolidate memory in a mouse model of Alzheimer’s disease. *Neurobiol. Learn. Mem.* 92 455–459. 10.1016/j.nlm.2009.05.001 19435612 PMC2772829

[B47] Parsons C. G. Stöffler A. Danysz W. (2007). Memantine: A NMDA receptor antagonist that improves memory by restoration of homeostasis in the glutamatergic system - too little activation is bad, too much is even worse. *Neuropharmacology* 53 699–723. 10.1016/j.neuropharm.2007.07.013 17904591

[B48] Peng Y. Jin H. Xue Y. H. Chen Q. Yao S. Y. Du M. Q. (2023). Current and future therapeutic strategies for Alzheimer’s disease: An overview of drug development bottlenecks. *Front. Aging Neurosci.* 15:1206572. 10.3389/fnagi.2023.1206572 37600514 PMC10438465

[B49] Power A. E. Berlau D. J. McGaugh J. L. Steward O. (2006). Anisomycin infused into the hippocampus fails to block “reconsolidation” but impairs extinction: The role of re-exposure duration. *Learn. Mem.* 13 27–34. 10.1101/lm.91206 16452651 PMC1360130

[B50] Prado-Alcalá R. A. Díaz, del Guante M. A. Garín-Aguilar M. E. Díaz-Trujillo A. Quirarte G. L. (2006). Amygdala or hippocampus inactivation after retrieval induces temporary memory deficit. *Neurobiol. Learn. Mem.* 86 144–149. 10.1016/j.nlm.2006.01.006 16540353

[B51] Przybyslawski J. Sara S. J. (1997). Reconsolidation of memory after its reactivation. *Behav. Brain Res.* 84 241–246. 10.1016/s0166-4328(96)00153-2 9079788

[B52] Puzzo D. Lee L. Palmeri A. Calabrese G. Arancio O. (2014). Behavioral assays with mouse models of Alzheimer’s disease: Practical considerations and guidelines. *Biochem. Pharmacol.* 88 450–467. 10.1016/j.bcp.2014.01.011 24462904 PMC4014001

[B53] Rai S. P. Krohn M. Pahnke J. (2020). Early cognitive training rescues remote spatial memory but reduces cognitive flexibility in Alzheimer’s disease mice. *J. Alzheimers Dis.* 75 1301–1317. 10.3233/JAD-200161 32417783 PMC7369118

[B54] Rogawski M. A. Wenk G. L. (2003). The neuropharmacological basis for the use of memantine in the treatment of Alzheimer’s disease. *CNS Drug Rev.* 9 275–308. 10.1111/j.1527-3458.2003.tb00254.x 14530799 PMC6741669

[B55] Rose S. P. (2000). God’s organism? The chick as a model system for memory studies. *Learn Mem*. 7, 1–17. 10.1101/lm.7.1.1 10706598

[B56] Rose S. P. Gibbs M. E. Hambley J. (1980). Transient increase in forebrain muscarinic cholinergic receptor binding following passive avoidance learning in the young chick. *Neuroscience* 5 169–178. 10.1016/0306-4522(80)90083-4 7366840

[B57] Rossato J. I. Radiske A. Gonzalez M. C. Apolinário G. de Araújo R. L. S. Bevilaqua L. R. M. (2023). NMDARs control object recognition memory destabilization and reconsolidation. *Brain Res. Bull.* 197 42–48. 10.1016/j.brainresbull.2023.03.013 37011815

[B58] Salinska E. (2006). The role of group I metabotropic glutamate receptors in memory consolidation and reconsolidation in the passive avoidance task in 1-day-old chicks. *Neurochem. Int.* 48 447–452. 10.1016/j.neuint.2005.11.015 16510211

[B59] Salinska E. Bourne R. C. Rose S. P. (2004). Reminder effects: The molecular cascade following a reminder in young chicks does not recapitulate that following training on a passive avoidance task. *Eur. J. Neurosci.* 19 3042–3047. 10.1111/j.0953-816X.2004.03407.x 15182312

[B60] Samartgis J. R. Schachte L. Hazi A. Crowe S. F. (2012). Memantine facilitates memory consolidation and reconsolidation in the day-old chick. *Neurobiol. Learn. Mem.* 97 380–385. 10.1016/j.nlm.2012.02.009 22425751

[B61] Sherry J. M. Crowe S. F. (2008a). Inhibition of cyclin-dependent kinase 5 by roscovitine impairs memory consolidation and reconsolidation in the day-old chick. *Pharmacol. Biochem. Behav.* 91 59–66. 10.1016/j.pbb.2008.06.010 18627776

[B62] Sherry J. M. Crowe S. F. (2008b). The non-NMDA receptor antagonist 6-cyano-7-nitroquinoxaline-2,3-dione (CNQX) impairs late reconsolidation of passive avoidance learning in the day-old chick. *Neurosci. Lett.* 442 244–248. 10.1016/j.neulet.2008.07.024 18640241

[B63] Sherry J. M. Milsome S. L. Crowe S. F. (2010). The roles of RNA synthesis and protein translation during reconsolidation of passive-avoidance learning in the day-old chick. *Pharmacol. Biochem. Behav.* 94 438–446. 10.1016/j.pbb.2009.10.006 19857511

[B64] Summers M. J. Crowe S. F. Ng K. T. (2003). Memory retrieval in the day-old chick: A psychobiological approach. *Neurosci. Biobehav. Rev.* 27 219–231. 10.1016/s0149-7634(03)00032-0 12788334

[B65] Tang B. C. Wang Y. T. Ren J. (2023). Basic information about memantine and its treatment of Alzheimer’s disease and other clinical applications. *Ibrain* 9 340–348. 10.1002/ibra.12098 37786758 PMC10527776

[B66] Tiunova A. A. Bezriadnov D. V. Anokhin K. V. (2024). Non-competitive NMDA antagonist MK-801 prevents memory reconsolidation impairment caused by protein synthesis inhibitors in young chicks. *Front. Pharmacol.* 15:1378612. 10.3389/fphar.2024.1378612 39027332 PMC11254664

[B67] Tiunova A. A. Bezryadnov D. V. Gaeva D. R. Solodovnikov V. S. Anokhin K. V. (2020). Memory reacquisition deficit: Chicks fail to relearn pharmacologically disrupted associative response. *Behav. Brain Res.* 390:112695. 10.1016/j.bbr.2020.112695 32407820

[B68] Webster S. J. Bachstetter A. D. Nelson P. T. Schmitt F. A. Van Eldik L. J. (2014). Using mice to model Alzheimer’s dementia: An overview of the clinical disease and the preclinical behavioral changes in 10 mouse models. *Front. Genet.* 5:88. 10.3389/fgene.2014.00088 24795750 PMC4005958

[B69] Zhang H. Jiang X. Ma L. Wei W. Li Z. Chang S. (2022). Role of Aβ in Alzheimer’s-related synaptic dysfunction. *Front. Cell. Dev. Biol.* 10:964075. 10.3389/fcell.2022.964075 36092715 PMC9459380

[B70] Zhao W. Q. Feng H. Bennett P. Ng K. T. (1997). Inhibition of intermediate-term memory following passive avoidance training in neonate chicks by a presynaptic cholinergic blocker. *Neurobiol. Learn. Mem.* 67 207–213. 10.1006/nlme.1997.3767 9159759

